# Role of *Clostridium perfringens* Necrotic Enteritis B-like Toxin in Disease Pathogenesis

**DOI:** 10.3390/vaccines10010061

**Published:** 2021-12-31

**Authors:** Kyung-Woo Lee, Hyun S. Lillehoj

**Affiliations:** 1 Animal Biosciences and Biotechnology Laboratory, Beltsville Agricultural Research Center, Agricultural Research Service, USDA, Beltsville, MD 20705, USA; hyun.lillehoj@usda.gov; 2 Department of Animal Science and Technology, Konkuk University, Seoul 05029, Korea

**Keywords:** *Clostridium perfringens*, necrotic enteritis, NetB toxin, pathogenesis, NE detection, broiler chickens

## Abstract

Necrotic enteritis (NE) is a devastating enteric disease caused by *Clostridium perfringens* type A/G that impacts the global poultry industry by compromising the performance, health, and welfare of chickens. Coccidiosis is a major contributing factor to NE. Although NE pathogenesis was believed to be facilitated by α-toxin, a chromosome-encoded phospholipase C enzyme, recent studies have indicated that NE B-like (NetB) toxin, a plasmid-encoded pore-forming heptameric protein, is the primary virulence factor. Since the discovery of NetB toxin, the occurrence of NetB^+^ *C. perfringens* strains has been increasingly reported in NE-afflicted poultry flocks globally. It is generally accepted that NetB toxin is the primary virulent factor in NE pathogenesis although scientific evidence is emerging that suggests other toxins contribute to NE. Because of the complex nature of the host-pathogen interaction in NE pathogenesis, the interaction of NetB with other potential virulent factors of *C. perfringens* needs better characterization. This short review will summarize the primary virulence factors involved in NE pathogenesis with an emphasis on NetB toxin, and a new detection method for large-scale field screening of NetB toxin in biological samples from NE-afflicted commercial broiler flocks.

## 1. Introduction

The world population is rapidly expanding and is expected to reach over nine billion people by the year 2050 [1]. Most of this population growth is expected to occur in Africa and Asia where the poultry industry will be required to sustainably meet the increasing demand for safer poultry meat and eggs. Thus, our role as animal scientists in combating world hunger is critical and there is a timely need to develop strategic priorities for sustainable food production systems. The global poultry industry is a dynamic industry that has successfully met an ever-increasing demand on global poultry meat and egg consumption. There are, however, ongoing challenges [2] to solve for sustainable poultry and eggs production. Those challenges include legislative bans on antibiotic use in animal feed, global climate change, and economically important disease outbreaks affecting the food industry, all of which could negatively affect optimum poultry performance. For example, it was estimated that a higher ambient temperature lowered the productivity and welfare of chickens resulting in an economic loss of USD128 to 165 million for the US poultry industry [3].

Over the last 50 years, sub-therapeutic doses of in-feed antibiotics have been a reliable tool to increase the welfare and productivity of chickens by controlling pathogenic bacteria and preventing dysbacteriosis. However, increasing concerns regarding the occurrence of antibiotic-resistant bacteria and drug residues in poultry meats halted the use of antibiotics in poultry feed globally. Finally, enteric disorders caused by *Eimeria* spp. or *Clostridium perfringens*, both of which are ubiquitous in the environment and gastrointestinal tract of animals, compromise the health, performance, and welfare of chickens. Coccidiosis caused by *Eimeria* spp. results in an economic loss of about 13 billion US dollars per year due to mortality and the use of in-feed anticoccidials [4]. Controlling enteric disorders is expected to be more challenging in the post-antibiotic era, and there is a timely need to develop novel solutions to promote the health and performance of chickens. Topics on gut health and alternatives to antibiotic use in chickens have been discussed elsewhere [2,5,6,7,8,9,10]. This review will focus on the devastating enteric disease that is mainly caused by avian-specific *C. perfringens.* Finding a sustainable solution to reduce economic losses caused by necrotic enteritis (NE) is a high priority for the global poultry industry as NE is a major challenge in antibiotic-free chicken production. This mini-review will summarize recent developments in understanding NE pathogenesis with an emphasis on the recently identified and putative principal virulence toxin, NE B-like (NetB) toxin. Future research directions to further understand NE pathogenesis will be briefly discussed.

## 2. Necrotic Enteritis

**Figure 1:**
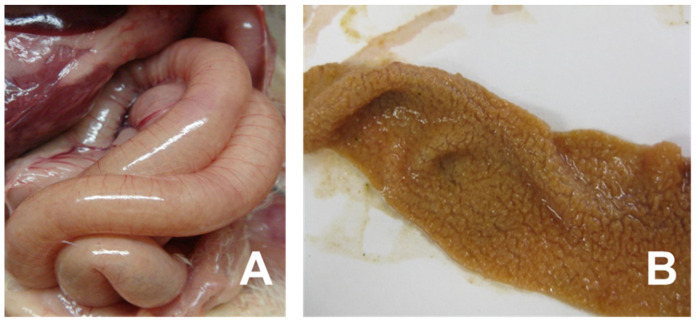
Clinical signs of necrotic enteritis in chickens. Panel (**A**) indicates small intestine showing friable, dilated and thin-walled filled with gas. Panel (**B**) shows jejunal/ileal mucosal exhibiting typical yellow pseudo-membrane, often called as Turkish towel appearance.

Necrotic enteritis (NE), an enteric disease in chickens caused by *Clostridium perfringens* type A/G, is a common disease that affects intensive broiler production globally. However, NE incidence has increased with the regulatory bans on in-feed antibiotics, which includes anticoccidials [11]. The reemergence of NE outbreaks in poultry production, especially in “No Antibiotics Ever (NAE)” poultry production operations, is increasingly prevalent. The NE causative agent, *C. perfringens,* is a ubiquitous bacterium in poultry housing and a common commensal in the chicken gastrointestinal tract [12]. NE often occurs in chickens between 2 and 6 weeks of age and mortality ranges from 2 to 50%. The severity of NE is classified as either clinical or subclinical with afflicted flocks exhibiting symptoms ranging from increased mortality (i.e., clinical NE) to decreased feed intake (i.e., subclinical NE) [13,14] in the afflicted flock. NE-afflicted chickens appear depressed and suffer from diarrhea with decreased performance, i.e., a low growth rate and feed intake with high feed conversion and uniformity. NE causes an acute enterotoxemia and the clinical signs last a short time, and often the only signs are a severe depression followed by a sudden increase in flock mortality [15]. Clinical NE is characterized by rapid death (less than 24 h) once NE clinical signs develop [16]. Cholangiohepatitis is associated with subclinical NE and is detected at the slaughterhouse where the animal is condemned [17]. The gross lesions at necropsy are primarily found in the small intestines, which may be ballooned, friable, and may have brown blood-tinged fluid with a foul odor. The mucosa of an NE-infected animal is usually covered with a tan to yellow pseudo-membrane described as a “Turkish towel” [11] (Figure 1). NE-related liver condemnations at the slaughterhouse can be as great as 20% [18]. The cost of NE to the global broiler industry has been estimated to be from USD two billion [17] to six billion [19] and is likely to increase as the global animal agricultural industry expands.

## 3. *Clostridium perfringens* Toxinotypes

*C. perfringens* is anaerobic, non-motile, Gram-positive spore-forming bacteria found ubiquitously, including as a normal microbiota in animals, and produces at least 20 virulent toxins and enzymes [20,21]. *C. perfringens* is a fast-growing bacterium with an 8–12 min generation time when cultured at 43 °C in optimal media that extends to 12–17 min at 37 °C. Once dominantly colonized under optimal environments (e.g., predisposing factors), the fast-growing *C. perfringens* can cause a rapid onset of NE [22]. Six virulent factors (e.g., toxins/enzymes) have been used to designate the strains from A to G depending on their production capability for α-toxin, β-toxin, ε-toxin, ι-toxin, enterotoxin, and NE toxin beta-like (NetB) toxin [21] (Table 1), and their functions and production have been reported [20,22,23]. Initially, *C. perfringens* had five toxinotypes based on the chromosomal presence of α-toxin, β-toxin, ε-toxin, or ι-toxin. However, the discovery of new toxin genes (e.g., *enterotoxin* and *netB*) and toxin-specific *C. perfringens*-mediated diseases has led to the classification of seven *C. perfringens* toxinotypes (Table 1). In the new toxinotyping scheme, *C. perfringens* type F produces α-toxin and enterotoxin but not β-toxin, ε-toxin, ι-toxin, or NetB toxin, and is an important enteric pathogen that causes food poisoning in humans [21,24]. This toxinotyping scheme emphasizes the role of enterotoxin production in *C. perfringens*-mediated food poisoning cases.

All toxinotypes (i.e., type A to G) of *C. perfringens* can extracellularly secrete high levels of α-toxin, which is encoded by the chromosomal *plc* gene (Table 1). α-Toxin is a zinc-metalloenzyme with a phospholipase C (plc) domain that is responsible for phospholipase, sphingomyelinase, and hemolytic activities [22]. Thus, α-toxin has long been considered a major virulence factor in NE necrotic lesions. However, the recently discovered NetB toxin by *C. perfringens* is now proposed to be the primary toxin responsible for NE pathogenesis in broilers [25]. Accordingly, *C. perfringens* type G is responsible for NE in chickens as it has genes encoding the two typing toxins α-toxin and NetB [21]. On the other hand, *C. perfringens* type A can only produce α-toxin, but not β-toxin, ε-toxin, ι-toxin, enterotoxin, or NetB toxin. One exception to the seven toxinotype scheme is one reported case that showed the *netB* gene in *C. perfringens* type C, which was isolated from a subclinical NE case [26]. Nonetheless, the *netB* gene is only detected in *C. perfringens* type G strains.

## 4. *C. perfringens* Toxins

*C. perfringens* proliferation inside the host requires the degradation of host tissues as it lacks many genes related to amino acid biosynthesis. Thus, the ability of *C. perfringens* to obtain essential nutrients for survival and growth depends on the many toxins and enzymes it produces. In addition to α-toxin and NetB toxin, *C. perfringens* type G also produces more than 20 toxins and enzymes that are responsible for NE pathogenesis (Table 2). *C. perfringens* type A/G has chromosome- or plasmid-borne genes that encode for α-toxin, perfringolysin O, collagenases, sialidases, hyaluronidases, collagen adhesion protein, NetB toxin, beta2 toxin, and TpeL toxin. These toxins and enzymes are currently recognized as being important, but more NE virulence factors are likely to be discovered [21].

*C. perfringens* toxins can be grouped into four categories [27] depending on their activities: (a) membrane damaging enzymes, (b) pore-forming toxins, (c) intracellular toxins, and (d) hydrolytic enzymes. Membrane damaging toxins such as α-toxin mainly damage the target cell membranes via their ability to hydrolyze host cell membrane constituents. The α-toxin (43 kDa), encoded by chromosome-borne *plc* gene, is 37 amino acid residues long with two domains, the N-terminal catalytic domain and the C-terminal membrane-binding domain [28]. Its mechanism of action is complex, requiring lipid hydrolysis (e.g., phosphatidylcholine and sphingomyelin) and plasma membrane binding via the *TrkA* receptor, which triggers a signal transduction pathway [29]. Synergistic interactions between α-toxin and sialidases secreted by *C. perfringens* have also been reported [29].

The pore-forming toxins is the largest class of bacterial protein toxins and includes perfringolysin O, NetB toxin, beta2 toxin, and enterotoxin. As the name indicates, pore-forming toxins can form either small or large pores through the membrane, a common mechanism of cell death. Perfringolysin O (or θ-toxin) is known to form pores by binding to cholesterol in the lipid bilayer of the membrane while enterotoxin is mediated by binding to claudin [30]. However, neither the NetB nor beta2-toxin receptors are known [30]. NetB is known to be a major virulence toxin in NE-afflicted chickens [39], but the role of beta2 toxin in chicken NE is controversial [44,45]. However, *C. perfringens* harboring *cpb2* gene has been known to play a role in *C. perfringens*-associated enteritis in piglets [46].

The third class is intracellular toxins that are internalized into host cells to disrupt the cellular cytoskeleton [27]. Those NE virulence candidates include the TpeL toxin, which is a member of the large clostridial toxin family. The TpeL toxin is proposed to potentiate the NE pathogenesis by *C. perfringens* type A/G strains [47]. TpeL has several domains that exhibit glycosyltransferase activity, autocatalytic activity, and a transmembrane domain that delivers the glycosyltransferase enzyme into the cytosol of host cells [41]. TpeL self-mediates host cell entry and mono-glycosylates Ras proteins, thus inhibiting Ras signaling and inducing cell apoptosis [43]. Gu et al. reported that five out of 19 *netB*-positive *C. perfringens* isolates from NE-afflicted chickens were also positive for *tpeL* and exhibited differing patterns of in vitro TpeL production [47]. Coursodon et al. showed that chickens infected with TpeL-positive *C. perfringens* exhibited a more rapid development of NE with a higher NE-associated mortality rate than those challenged with TpeL-negative *C. perfringens* [42]. These finding suggests that the TpeL toxin can potentiate the virulence effect of NE-inducible *C. perfringens* strains [42].

Finally, hydrolytic enzymes degrade or hydrolyze substrates associated with the surface of the host cells and include both polysaccharide-degrading enzymes (e.g., sialidases and hyaluronidases) and proteinases (e.g., collagenases). Collagens and hyaluronic acids are the predominant fibrous proteins composing the extracellular matrix. Sialic acid-containing glycoconjugates cover the outer surface of the cytoplasmic membrane, some participate in connecting to the extracellular matrix components or in cell-cell interactions [48]. Hydrolytic toxins are believed to function as virulence factors that aid *C. perfringens* proliferation and spread in infected tissues and possibly potentiate other toxins by facilitating their diffusion [48]. The *C. perfringens* genome encodes five hyaluronidase genes designated as *nagH*, *nagI*, *nagJ*, *nagK*, and *nagL* (Table 2) [35]. Sialidases (or neuraminidases) function to degrade sialic acids from glycoconjugates found throughout the body and making them available as nutrients to *C. perfringens* [34,49]. *C. perfringens* produces three different sialidases (designated NanH, NanI and NanJ) with NanI and NanJ being extracellularly secreted. The non-secreted NanH acts on partially degraded carbohydrate polymers that are transported into the bacterial cells. Trypsin can enhance the activity of NanI but not NanH or NanJ [49]. Collagenase (or kappa toxin), encoded by *colA*, is a primary *C. perfringens* toxin that degrades collagen, an integral component of the host connective tissues and basal membranes. Collagen disruption by collagenase may lead to the loss of tissue integrity and subsequent tissue necrosis [36]. Findings that collagenase activity was increased in the intestinal tissue of broilers challenged with *C. perfringens* may suggest its involvement as a virulent factor in the initial stages of NE [50].

In addition to the toxins and enzymes that we discussed above, collagen adhesion protein, encoded by *cnaA,* has recently been reported in NE-producing *C. perfringens* strains [37]. *C. perfringens* strains harboring *cnaA* were all virulent and bound to collagen type IV, V, and gelatin in vitro. In a follow-up study by Wade et al., *cnaA*-deleted *C. perfringens* lost the ability to bind to collagen in vitro, did not induce NE lesions in vivo, and showed a reduced ability to colonize on the ileal mucosa in vivo [51]. These results show the importance of *C. perfringens* adherence to extracellular matrix proteins as a crucial virulence factor in NE pathogenesis [51].

## 5. *C. perfringens* NetB Toxin and Its Role in NE

Recent molecular and proteomic studies have increased our understanding of the structure and function of NetB in NE pathogenesis [33,38,52,53,54]. The *netB* gene is encoded in the 42-kb NELoc-1 region on an ca. 85-kb plasmid [55]. NetB toxin production is tightly controlled and regulated by the VirR/VirS two-component signal system [23] and an accessory agr-like quorum sensing system [54] located on the *C. perfringens* chromosome. NetB is secreted as 33-kDa β-barrel toxin that forms 1.6- to 1.8-nm pores in susceptible cells. It shares some structural similarity (less than 40%) with β-toxin from *C. perfringens*, α-hemolysin, γ-toxin, and leukocidin from *Staphylococcus aureus*, and cytotoxin K from *Bacillus cereus* [25,39]. The NetB toxin is cytotoxic against the chicken leghorn male hepatoma cell line (LMH) but did not affect the viability of chicken embryo fibroblast cells (DF-1) or a chicken macrophage cell line (HDll) [39]. Keyburn et al. clearly showed that the NetB toxin, but not alpha toxin, is an essential virulence factor for NE in broiler chickens using *plc*- and *netB*-deletion mutants of the virulent *C. perfringens* EHE-NE18 strain [39,56]. Furthermore, Coursodon et al. reported that a *plc*-deletion mutant of the virulent *C. perfringens* JGS4143 strain induced NE in chickens [57]. However, the latter also detected α-toxin in chickens challenged with the *plc*-deletion mutant. In addition, Zhou et al. developed a *C. perfringens* strain missing the NELoc-1 virulence plasmid from the virulent *C. perfringens* CP1 strain, which was then complemented with *netB*. While the virulent *C. perfringens* strain CP1 induced NE, the CP1 strain missing the NELoc-1-encoding plasmid did not [58]. However, complementing the *netB*-deficient CP1 variant strain with *netB* gene restored its intermediate virulence. Collectively, these studies [39,56,57,58] proved that other virulence factors on the NELoc-1 plasmid or elsewhere, are required for NE in addition to α-toxin and NetB since NE disease has been induced in broiler with both *netB*-positive and -negative *C. perfringens* isolates.

The molecular-based detection of *netB*-positive *C. perfringens* isolates in NE-afflicted broiler flocks has been reported over the last decade (Table 3) [59,60,61,62,63,64,65]. A summary of the most frequently studied virulent NE-causing strains globally highlights the importance of *netB^+^ C. perfringens* for experimentally reproducible NE models (Table 3). In contrast, findings on the presence of *netB*-positive *C. perfringens* strains in healthy chickens [60,61,62,64,66,67], the recovery of *netB*-negative strains from NE-afflicted chickens [59,60,61,63,67,68], and successful NE reproduction by *netB*-negative *C. perfringens* [39,68,69] indicate the role of additional virulence factors in NE pathogenesis.

There is limited understanding of NE pathogenesis in the field although *C. perfringens* overgrowth often causes the simultaneous and dynamic development of NE lesions in chickens susceptible to NE [70,71,72]. Barbara et al. reported that NE-causing *C. perfringens* are the dominant colonizer in the chicken gut compared to non-NE-causing strains due to the secretion of inhibitory antimicrobial bacteriocins and/or toxins [73]. Necrotic lesions caused by *netB*-positive *C. perfringens* start at the basal membrane and lateral segments of the enterocytes then spreads to the entire lamina propria [71,72]. In addition, the gut integrity is weakened by hydrolytic enzymes including collagenases, sialidases, and hyaluronidases while cell surface *C. perfringens* proteins facilitate its binding to the damaged intestinal mucosa, leading to severe NE lesions [70,74].

## 6. Incidence of *NetB*-Positive *C. perfringens* Isolated from Broilers

Since the discovery of the NetB toxin as an essential virulence factor in NE, the prevalence of *netB*-positive *C. perfringens* isolates in NE disease outbreaks has been reported (Table 4). In general, culture methods utilizing media and agar were used to isolate *C. perfringens* from chicken digesta or litter samples prior to the *netB* genotyping with polymerase chain reaction (PCR) or quantitative PCR. *C. perfringens* can be directly isolated by plating gut or environmental samples on selective agar (e.g., TSC agar) or by a liquid culture (e.g., FTG, CMM, or BHI) followed by plating on solid agar (e.g., TSC, blood, or BHI). The function of selective media for *C. perfringens* has been documented elsewhere [71] although their contributions to growth and toxin production have not studied.

Several conclusions can be drawn from studies that describe *C. perfringens* isolation from NE-afflicted or healthy chickens (Table 4): (1) Field NE occurs globally, (2) *netB*-positive *C. perfringens* are isolated from both NE-afflicted and healthy chickens, (3) *netB*-positive *C. perfringens* is more abundant in NE-afflicted vs. healthy chickens, (4) *netB*-positive *C. perfringens* may not be isolated from confirmed NE clinical cases, (5) *netB*-negative *C. perfringens* can be isolated from NE-afflicted chickens, and (6) *netB*-positive *C. perfringens* can be detected in retail broiler meats. Thus, these findings indicate that NE is a complex disease caused by multiple contributing and predisposing factors (e.g., *C. perfringens* toxins, *Eimeria*, stress, nutrition, or environment) although the NetB toxin is considered to be the primary virulent factor.

## 7. In Vitro and In Vivo NetB Production by *NetB*-Positive *C. perfringens* Isolates

Until recently, native NetB protein was only detected in vitro in culture supernatants from *netB*-positive *C. perfringens* strains by Western blot analysis using polyclonal anti-recombinant NetB antiserum [39,53,60,85] or a cell-based cytotoxicity assay [39,94]. Abildgaard et al. showed that 12 out of 13 *C. perfringens* isolates from NE chickens were NetB positive whereas only 4 out of 14 *C. perfringens* strains from healthy chickens produced NetB [60]. This study also emphasized that *C. perfringens* isolated from NE-afflicted chickens secreted much higher amounts of NetB toxin in vitro compared to those from normal chickens. Allaart et al. reported that 9 out of 43 *netB*-positive *C. perfringens* strains isolated from hens with subclinical NE produced NetB protein in vitro [26]. There are no reports showing in vitro NetB production by *netB*-negative *C. perfringens*. In one study, all 22 *netB*-positive *C. perfringens* strains isolated from chickens with clinical NE produced NetB [85]. These studies collectively indicate that not all *netB*-positive *C. perfringens* may secrete NetB in vitro. The conflicting results may be related to the difference in *netB* gene copy number among *netB*-positive *C. perfringens* isolates. Indeed, Gu et al. isolated 18 *netB*-positive *C. perfringens* from NE-afflicted chickens and showed that only half of the *netB*-positive *C. perfringens* strains had higher *netB* gene copy numbers relative to their 16S rDNA levels [47]. Gu’s observation [47] suggests that the presence of the *netB* gene in *C. perfringens* does not guarantee the secretion of the NetB toxin in vitro.

A detection method to measure native NetB protein in biological samples (e.g., from NE-afflicted or healthy chickens) is not currently available. With increasing restriction of antibiotic-free poultry production, there is a need to develop a high throughput, reliable analytical tool to detect native NetB protein in biological samples to adequately address several questions related to fundamental and applied research on NE: (1) whether in vitro and in vivo production of NetB protein by *netB*-positive *C. perfringens* is related, (2) whether in vivo NetB protein can be detected by *netB*-negative *C. perfringens* in NE models, and (3) whether routine screening of biological samples (feces, litters, tissues, or sera) can predict virulent *C. perfringens* NE outbreaks on farms for timely management. The recent development of a NetB antigen-specific ELISA system [95] to detect and quantify NetB in biological samples should enhance our ability to manage field NE outbreaks. Lee et al. developed multiple NetB-specific monoclonal antibody pairs for capture ELISA to detect *Escherichia coli* NetB protein and native NetB protein secreted by *netB*-positive *C. perfringens* isolates [95]. Furthermore, the availability of a NetB-specific ELISA will make it possible to quantify the NetB toxin in vitro and in vivo. For example, Keyburn et al. showed that 1:16-diluted culture supernatant of *netB*-positive *C. perfringens* exhibited cytopathic effects in LMH cells in vitro [39]. In addition, Keyburn et al. found that purified NetB protein ranging from 1.89 to 242.4 nM (calculated from 8 µg to 62.5 ng/well in 24-well format) was cytotoxic to LMH cells [39]. Similarly, Savva et al. observed that purified NetB induced morphological damage in LMH cells [38]. However, Savva et al. used much higher NetB concentrations ranging from 62.5 to 4000 nM (calculated from 412.5 ng to 26.4 µg/well in 96-well format) in a cytotoxicity assay with a median cytotoxic dose of 800 nM [38]. Until recently, it is not known whether those cytotoxic concentrations are biologically relevant to NE development in vivo. Lee et al. for the first time detected and reported the approximate concentration of NetB toxin to be 2.23 nM in the gut digesta from NE-afflicted broiler chickens [96]. The latter study [96] concluded that low NetB toxin concentrations used in vitro studies [39] are likely to be biologically relevant to induce NE development in vivo.

## 8. Concluding Remarks

NE is not only an emerging threat to the global broiler industry but also compromises the welfare and health of commercial poultry in the era of antibiotic-independent poultry production [12,97]. This threat will be enhanced under the “No Antibiotics Ever (NAE)” and “Antibiotic-free” (ABF) broiler production systems. Thus, understanding NE pathogenesis and developing sensitive detection assay are important to reduce economic losses due to NE and to develop early management strategies against NE. With the development of molecular and proteomic technologies, the NetB toxin has been regarded as the primary virulence factor in NE pathogenesis. However, new scientific evidence is emerging to implicate other *C. perfringens* toxins and enzymes that can potentiate NE pathogenesis with or without NetB. Although the presence of *netB* in *C. perfringens* isolates can be typed with PCR and the NetB protein can be detected using Western blots, there is a need for a more defined large-scale immunoassay that can detect toxins associated with NE pathogenesis in the field samples from NE-afflicted farms. Recent availability of NetB-specific mouse monoclonal antibodies and a high throughput ELISA detection system will now allow sensitive NetB detection in samples from poultry farms. These NetB-specific monoclonal antibody-based detection assays will be a valuable tool in addressing fundamental questions of host-pathogen immunobiology in NE and as a screening tool for validating the efficacy of novel alternative-to-antibiotics feed additives or vaccines to mitigate the negative effects of NE.

## Figures and Tables

**Table 1:** Revised toxin-based classification of *Clostridium perfringens*.

Type	Toxin ^A^ Produced (Structural Gene)					
	α-Toxin (*cpa*)	β-Toxin (*cpb*)	ε-Toxin (*etx*)	ι-Toxin (*iap*)	CPE (*cpe*)	NetB (*netB*)
A	+	−	−	−	−	−
B	+	+	+	−	−	−
C	+	+	−	−	±	−
D	+	−	+	−	±	−
E	+	−	−	+	±	−
F	+	−	−	−	+	−
G	+	−	−	−	−	+

^A^ Symbol (+) indicates the presence of the toxin and symbol (−) indicates the absence of the toxin.

**Table 2:** Properties of *Clostridium perfringens* toxins.

Full Name	Gene	Other Name	Gene Location ^A^	Size (kDa)	Activity	References
Alpha-toxin	cpa, plc	phospholipase C	C	43	To hydrolyze cell membrane phospholipids	[22]
Perfringolysin O	pfoA	θ-toxin (pore-forming toxin)	C	54	Pore formation via binding to cholesterol-comprising cell membrane	[22,30]
Collagenase	colA	κ-toxin	C	120	To degrade collagen that is main component of connective tissues of the host cells	[22,31]
Sialidase	nanI	secreted major neuramidases	C	77	Involved in removal of sialic acids from a variety of glycoconjugates on cell membranes	[32,33,34]
Sialidase	nanH	non-secreted neuramidases	C	43		
Sialidase	nanJ	secreted neuramidases	C	129		
Hyaluronidase	nagH	µ-toxin	C	≈182	To degrade hyaluronan coating cells allowing direct contact between pathogen and host cells, or to degrade hyaluronan leading to viscosity reduction facilitating increased permeability of the connective tissues	[35,36]
Hyaluronidase	nagI	µ-toxin	C	≈146		
Hyaluronidase	nagJ	µ-toxin	C	≈128		
Hyaluronidase	nagK	µ-toxin	C	≈131		
Hyaluronidase	nagL	µ-toxin	C	≈127		
Collagen adhesion protein	cnaA	cell surface protein	C	78	Binding of the *Clostridium perfringens* to collagen types IV, V and gelatin	[37]
NE B-like toxin	netB	pore-forming toxin	P	33	To form heptameric, hydrophilic pores with a central diameter of approximately 26 Å	[38,39]
Beta2 toxin	cpb2	pore-forming toxin	P	28	Pore forming leading to cell disruption	[40]
Toxin *C. perfringens* large cytotoxin	tpeL	large clostridial toxin	P	≈205	Ras-specific glucosyltransferase activity inactivating the Ras signaling pathway leading to apoptosis	[41,42,43]

^A^ C = chromosome; P = plasmid.

**Table 3:** NE strains often used in experimental NE studies.

Strain	Country	Status	Virulent Genes					References
			Plc	NetB	CnaA	TpeL	Cpb2	
Del1	USA	NE ^A^	+	+	+	−	+	[47,68]
TpeL17	USA	NE	+	+	na ^B^	+	+	[47]
N11	USA	Health	+	−	−	−	+	[68,75]
CP15	USA	NE	+	−	−	−	−	[68,75]
JGS4143	USA	NE	+	+	na	−	+	[41,76]
CP-6	USA	NE	+	+	na	na	na	[77,78]
EHE-NE18	Australia	NE	+	+	+	−	+	[37,39,75,79,80]
WER-NE36	Australia	NE	+	+	−	−	+	[37,75,79,80]
CP1	Canada	NE	+	+	na	na	+	[52,81]
Strain 56	Belgium	NE	+	+	+	na	−	[82,83]
S48	Denmark	NE	+	+	na	na	na	[60,84]

^A^ NE = necrotic enteritis. ^B^ na = not available.

**Table 4:** Incidence of necrotic enteritis (NE) B-like toxin gene in *Clostridium perfringens* G isolates from retail chicken meats or chickens afflicted with or without clinical or subclinical NE.

Country	Study Year ^A^	Detection Method	NE Chicken, *n*/Total		Healthy Chicken, *n*/Total		Ref.
			*NetB* Positive	%	*NetB* Positive	%	
Australia	2010	PCR	14/18	77.8	-	-	[25]
Australia/Canada/Belgium/Denmark	2010	PCR	31/44	70.5	2/55	3.6	[85]
Brazil	2012	PCR	0/22	0.0	-	-	[86]
Canada	2005–2007	PCR	39/41	95.1	7/20	35.0	[87]
Canada	2011–2012	PCR	9/45	20.0	12/18	66.7	[62]
Canada	2011–2012	PCR	41/41	100.0	26/30	86.7	[62]
Canada	2010	PCR	6/6	100.0	4/5	80.0	[66]
Canada ^B^	2010	PCR	-	-	39/183	21.3	[88]
Denmark	1997–2002	PCR	13/25	52.0	14/23	60.9	[60]
Denmark/ Finland	1997–2001	PCR	12/22	54.5	0/8	0.0	[89]
Iran	2016	PCR	8/45	17.8	-	-	[90]
Italy ^C^	2015–2017	qPCR	-	-	31/151	20.5	[65]
Italy	2009	PCR	16/30	53.3	4/22	18.2	[91]
Korea	2010–2012	PCR	8/17	47.1	2/50	4.0	[61]
Korea ^B^	2018	PCR	-	-	4/9	44.4	[92]
Netherlands	2012	PCR	43/45	95.6	-	-	[26]
Sweden	2004	PCR	31/34	91.2	- ^D^	25.0	[93]
Sweden	2004	PCR	16/23	69.6	-	-	[93]
Sweden	2004	PCR	0/11	0.0	-	-	[93]
USA	2004–2009	PCR	17/20	85.0	10/54	18.5	[67]
USA	2009	PCR	7/12	58.3	7/80	8.8	[59]
USA	2018	qPCR	11/15	73.3	9/15	60.0	[63]
USA	2016	PCR	119/145	82.1	59/85	69.4	[64]
USA	2003–2004	PCR	19/19	100.0	-	-	[47]

^A^ = year of publication was provided unless the studied year was not indicated. ^B^ = retail chicken meats. ^C^ = flock surveillance with no health status. ^D^ = not specified.

## Data Availability

Not applicable.
